# The complete chloroplast genome of *Spartina alterniflora*

**DOI:** 10.1080/23802359.2020.1776173

**Published:** 2020-06-12

**Authors:** Yang Zhao, Kai Wang, Yingying He, Yanfeng Wang, Changfeng Qu, Jinlai Miao

**Affiliations:** a First Institute of Oceanography, Ministry of Natural Resource, Qingdao, Shandong, China; b College of Marine Science and Biological Engineering, Qingdao University of Science and Technology, Qingdao, Shandong, China; c Laboratory for Marine Drugs and Bioproducts of Qingdao National Laboratory for Marine Science and Technology, Qingdao, Shandong, China

**Keywords:** *Spartina alterniflora*, chloroplast genome, phylogenetic analysis

## Abstract

*Spartina alterniflora* (also named as *Sporobolus alterniflorus*) grows in coastal salt marshes area, which has important economic value in coastal natural wetlands. In the process of this research, the whole chloroplast genome sequence of *Spartina alterniflora* was recovered by Illumina sequencing. The complete genome was 135,560 bp in length with 38.45% GC content which was a circular genome containing a large single-copy region (LSC, 80,828 bp), a small single-copy region (SSC, 12,714 bp) and a pair of inverted repeat regions (IRs, 42,018 bp). Totally, it encodes 130 genes, including 84 protein-coding genes, 38 tRNAs, and 8 rRNAs. Phylogenetic analysis indicated that *Spartina alterniflora* was closely related to *Sporobolus maritimus*.

*Spartina alterniflora* (also named as *Sporobolus alterniflorus*), which grows in salt marshes along the coast, is a dominant species belonging to Poaceae family. It was initially introduced to China from the coast of North America for coastal protection purposes in 1979 (Wan et al. 2009). Most importantly, *Spartina alterniflora* has mechanisms of salt tolerance and subsequently is used to explore its genome information for crops productivity (Subudhi and Baisakh 2011). It is adapted to highly saline environments where only halophytic plants can grow well (Zhang et al. 2017). Herein, the complete chloroplast genome of *Spartina alterniflora* we provided may be used for further studying salt tolerant mechanisms of Poaceae.

The fresh leaves of *Spartina alterniflora* were collected from beach of Zhejiang Qinshan Nuclear Power (30°N, 121°E), and the specimen (Accession no. FIO2019301212) was maintained in the Key Laboratory of Marine Bioactive Substances, the First Institute of Oceanography, Ministry of Natural Resource, China. The whole genome was sequenced with the Illumina NovaSeq PE150 at the Beijing Novogene Bioinformatics Technology Co., Ltd. Here, the chloroplast genome of *Spartina alterniflora* was assembled by the SPAdes software (Bankevich et al. 2012). The circular chloroplast genome was annotated with the DOGMA (Lohse et al. 2013). The descripted genomic sequence has been submitted to GeneBank with the accession numberMT311317.

The circular genome chloroplast of *Spartina alterniflora* was 135,560 bp in size. The large single copy region (LSC, 80,828 bp) and the small single copy region (SSC, 12,714 bp) were separated by a pair of reverse repeat regions (IRs, 42,018 bp). There are 130 genes in the chloroplast genome containing 84 protein-coding genes, 38 tRNA genes and 8 rRNA genes. The overall GC content of the genome accounted for38.45%.

To figure out the phylogenetic placement within the family Poaceae, the phylogenetic analysis was performed using MEGA 7.0 (Sudhir et al. 2016) with 1000 bootstrap replicates based on other 14 species complete genome sequences of Poales recorded in Genbank of NCBI database. The result demonstrated that *Spartina alterniflora* was closely related with *Sporobolus maritimus* (Figure 1).

**Figure 1.**
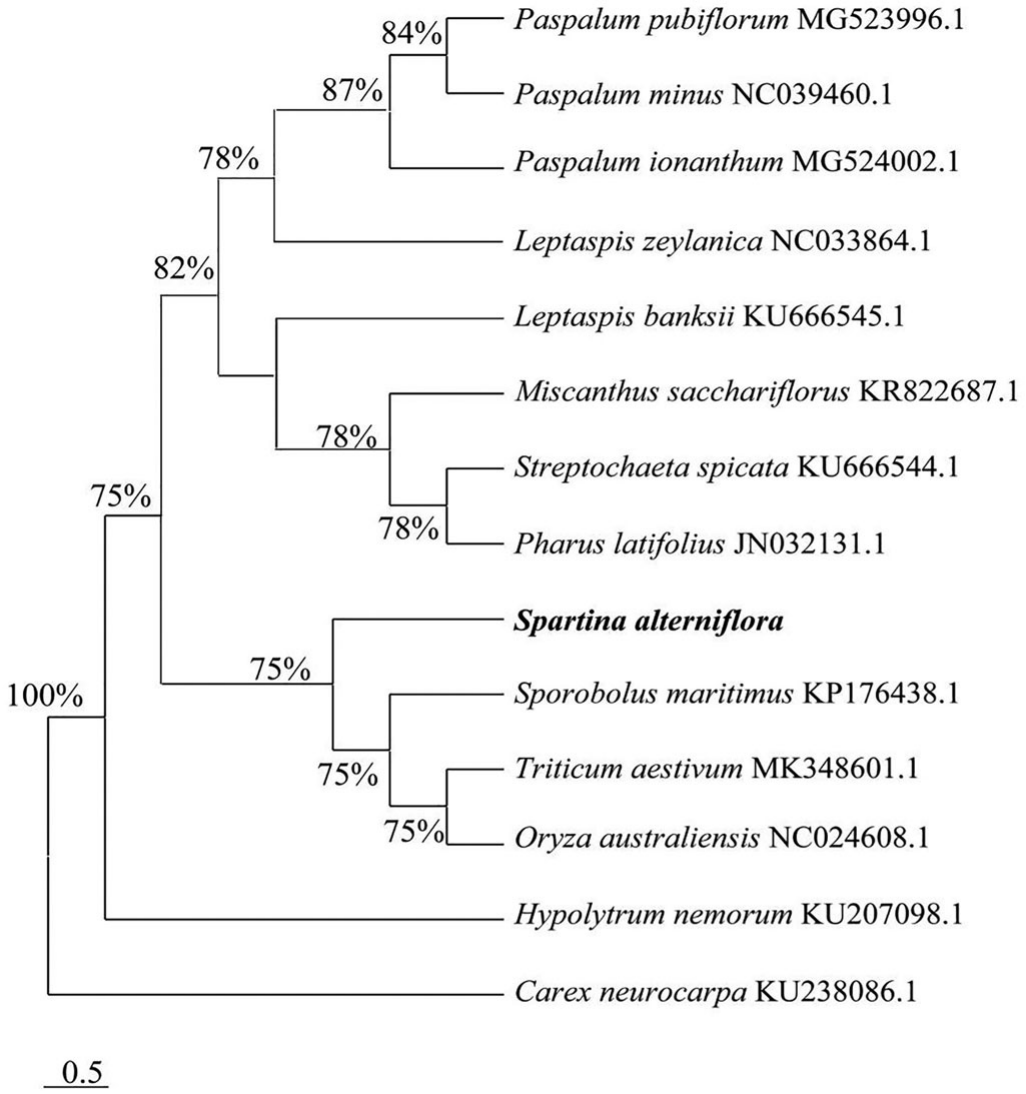
Neighbor-Joining phylogenetic tree based on 15 complete chloroplast genomes.

## Data Availability

The data that support the findings of this study are openly available in the NCBI at https://www.ncbi.nlm.nih.gov/, reference number of MT311317.
